# IL-33 Deficiency Attenuates Lung Inflammation by Inducing Th17 Response and Impacting the Th17/Treg Balance in LPS-Induced ARDS Mice via Dendritic Cells

**DOI:** 10.1155/2022/9543083

**Published:** 2022-12-16

**Authors:** Li Cheng, Yang Jiao, Wei Jiang, Xin Zhang, Liping Zhang, Gongwei Jia

**Affiliations:** ^1^Department of Health Management Center, The Second Affiliated Hospital of Chongqing Medical University, 76 Linjiang Road, Yuzhong District, Chongqing 400010, China; ^2^Department of Respiratory, The Second Affiliated Hospital of Chongqing Medical University, 76 Linjiang Road, Yuzhong District, Chongqing 400010, China; ^3^Department of Rehabilitation, The Second Affiliated Hospital of Chongqing Medical University, 76 Linjiang Road, Yuzhong District, Chongqing 400010, China; ^4^Department of Rehabilitation, Hubei College of Chinese Medicine, 87 Xueyuan Road, Jingzhou, Hubei Province 434020, China

## Abstract

**Objectives:**

The characteristic pathophysiological feature of acute respiratory distress syndrome (ARDS) is a dysregulated inflammatory response. T helper 17 (Th17) cells in the lung are inflammatory cells that contribute to pulmonary inflammatory cascades. In addition, Th17/regulatory T cells (Treg cells) also play an important role in the inflammatory process. Dendritic cells (DCs) can regulate the differentiation of CD4+ T cells, including Th17 and Treg cells. Recent evidence revealed that interleukin-33 (IL-33) signaling could activate and mature DCs. Therefore, the aim of this study was to investigate the effects of IL-33 on inflammation and immunoregulation by inducing the Th17 response and influencing the Th17/Treg balance in LPS-induced ARDS.

**Methods:**

IL-33 gene knockout mice and the administration of recombinant mouse IL-33 (rmIL-33) were used to investigate the role of IL-33 and the underlying mechanisms in an LPS-induced ARDS model. Hematoxylin and eosin (H&E) staining, wet/dry (W/D) weight ratios, cell counts, and the levels of tumor necrosis factor-*α* (TNF-*α*), interleukin-1*β* (IL-1*β*), interleukin-6 (IL-6), interleukin-17 (IL-17), and interleukin-10 (IL-10) in bronchoalveolar lavage fluid (BALF) were investigated. The levels of IL-33, orphan nuclear receptor gamma t (ROR*γ*t), and forkhead transcription factor protein 3 (FOXP3) protein in lung tissue were evaluated by Western blotting. The mRNA expression levels of IL-33 and ROR*γ*t were measured by quantitative real-time polymerase chain reaction (qRT-PCR). Th17 and Treg cell frequencies were determined by flow cytometry. The levels of IL-6 in the supernatant in a dendritic cell culture system were examined by ELISA.

**Results:**

Increased expression of IL-33 was observed in mice with LPS-induced ARDS. IL-33 deficiency significantly inhibited inflammation and attenuated LPS-induced ARDS, whereas pretreatment with rmIL-33 aggravated pulmonary inflammatory response. Furthermore, depletion of IL-33 inhibited Th17 cells, significantly decreased ROR*γ*t mRNA and protein expression and IL-17 levels in BALF, and led to less differentiation of T cells into Th17 cells than Treg cells. Moreover, IL-33^−/−^ DCs secreted less IL-6 and IL-23 than normal control DCs.

**Conclusion:**

IL-33 deficiency alleviated lung injury in the LPS-induced ARDS model, which was closely related to suppressing Th17 responses and regulating the Th17/Treg balance. The expansion of Th17 cells and imbalance in Th17/Treg cells may be associated with IL-6 and IL-23 secreted from IL-33-activated DCs.

## 1. Background

Acute respiratory distress syndrome (ARDS) is a type of acute, diffuse, inflammatory lung injury that leads to increased pulmonary vascular permeability, increased lung weight, and the loss of aerated lung tissue [1]. The typical pathophysiological feature of ARDS is dysregulated inflammation. Although inflammation is an important defensive response that eliminates damaging agents [2], excessive inflammation can be dangerous, as it can result in cell, tissue, and organ injuries [3]. Fundamental to lung inflammation in ARDS is the body's immune response to a variety of local and systemic stimuli. The mechanisms by which the immune system performs its tasks efficiently remain the focus of ongoing investigations. T helper 17 (Th17) cells, which are a subpopulation of CD4+ T lymphocytes, are involved in the pathogenicity and immunoprotective mechanisms in various autoimmune diseases, such as inflammatory bowel disease, multiple sclerosis, and rheumatoid arthritis [4–6]. In addition, emerging evidence has shown that Th17 cells play a critical role in the production and induction of proinflammatory cytokines during infections [7]. In particular, clinical trials suggested that high levels of CD4+ T cell activation and proliferation, along with the presence of Th17 cells, may contribute to the pathogenesis of ARDS by mediating lung inflammation [8, 9]. We previously demonstrated that Th17 cells were significantly activated in the early stage of ARDS and that suppressing the Th17 response could attenuate ARDS [10].

Regulatory T (Treg) cells, which can also be activated in ARDS and resolve acute lung injury by correcting the elevated levels of proinflammatory cytokines and neutrophil apoptosis [11], share reciprocally regulated developmental pathways with Th17 cells. In particular, the ratio of Th17/Treg cells is a novel risk factor in patients with early ARDS [9].

Interleukin-33 (IL-33), a nuclear cytokine in the IL-1 family, functions as an “alarmin” that is released from dead cells and exerts proinflammatory or protective effects during various infections. IL-33 binds to specific receptors known as suppression of tumorigenicity 2 (ST2) to exert its biological functions [12]. IL-33 can induce the activation and maturation of dendritic cells (DCs) [13], and mature DCs can present antigens to naïve CD4+ T cells to mediate their differentiation to effector T cells. It has also been reported that ST2 is expressed in Treg cells in the lung [14] and that IL-33 can inhibit Treg cell differentiation and promote the conversion of stable Treg cells to Th17 cells [15]. Thus, we hypothesized that IL-33 might play vital roles in ARDS by promoting and sustaining inflammation and tissue injury.

The aim of this study was to investigate the effect and underlying mechanism of IL-33 in LPS-induced experimental ARDS. We explored the importance of IL-33 in accelerating the expansion of Th17 cells, promoting their activity, and impacting the balance of Th17/Treg cells to mediate uncontrolled inflammation during ARDS.

## 2. Materials and Methods

### 2.1. Ethics Statement

All animal procedures in this study were approved by the Ethics Committee of Chongqing Medical University and were performed according to the instructions of the National Institutes of Health Guide for the Care and Use of Laboratory Animals.

### 2.2. Mice and Experimental Protocol

Wild-type (WT) C57BL/6 male mice aged 6–8 weeks were purchased from the Laboratory Animal Center of Chongqing Medical University, and IL-33 knockout (IL-33^−/−^) mice (NM-KO-190436) on a C57BL/6 background were purchased from Shanghai Model Organisms Center (Shanghai, China). All mice were housed under specific pathogen-free conditions with free access to water and chow. After anaesthetization with an intraperitoneal injection of sodium pentobarbital (50 mg/kg), the mice were subjected to intratracheal (i.t.) instillation of 3 mg/kg lipopolysaccharide (LPS, *Escherichia coli* serotype O111: B4; Sigma-Aldrich, St. Louis, MO, USA) dissolved in sterile phosphate-buffered saline (PBS). A sham operation was performed in a similar manner with the same volume of PBS instead of LPS. For IL-33 injection experiments, 1000 ng of recombinant mouse IL-33 (rmIL-33) per mouse was dissolved in sterile PBS (rmIL-33, 3236-ML-010; R&D Systems) and injected intraperitoneally following intratracheal instillation of PBS [16]. Control mice received the same volume of PBS at the same time point. After 2 days of LPS insult, the mice were sacrificed for further analysis according to the Interdisciplinary Principles and Institutional Animal Care and Use Committee guidelines.

### 2.3. Assessment of Lung Edema

Left lung tissue was removed, weighed, and dried in an oven at 55°C for 72 hours. The dry weight was then measured, and the wet/dry (W/D) ratio was calculated.

### 2.4. Histopathological Analysis

The middle lobe of the right lung was collected and fixed in 4% paraformaldehyde, embedded in paraffin, cut into 5 *μ*m sections, stained with hematoxylin and eosin (H&E), and measured by optical microscopy. Histological evaluations were performed based on ten randomly selected high-power fields (×200) in each section. Lung injury histopathological scores were calculated according to a previous study [17].

### 2.5. Bronchoalveolar Lavage Fluid (BALF)

Bronchoalveolar lavage was collected by flushing 1 ml of sterile normal saline back and forth three times through a trimmed 18-G catheter tracheal cannula as previously described [18]. After centrifugation at 500 × *g* for 20 minutes at 4°C, protein concentrations in BALF supernatants were measured by a bicinchoninic acid protein assay (BCA) kit (Solarbio Technology Co., Ltd., Beijing, China).

### 2.6. ELISA

Serum was collected from blood and prepared for ELISA analysis according to a previous study [19]. The levels of tumor necrosis factor-*α* (TNF-*α*), interleukin-1*β* (IL-1*β*), interleukin-6 (IL-6), interleukin-17 (IL-17), and interleukin-10 (IL-10) in BALF or mouse serum were measured by commercially available ELISA kits (Beyotime, China) according to the manufacturer's instructions.

### 2.7. RNA Isolation and Quantitative Real-Time PCR (qRT-PCR)

According to the manufacturer's instructions, total RNA was extracted from lung homogenates using RNAiso Plus (Takara Bio Inc., Japan). Then, total RNA was reverse transcribed to cDNA using the PrimeScript RT Reagent kit (Takara Bio Inc., Japan). Real-time quantitative PCR was performed using TB Green Premix Ex Taq II (Takara Bio Inc.) according to the manufacturer's instructions. The relative expression levels of target genes were quantified using the comparative threshold cycle (Ct) method (2^−ΔΔCt^). The primer sequences are shown in Table 1.

### 2.8. Western Blot Analysis

The samples were lysed in RIPA buffer, and protein levels were quantified using a BCA protein assay kit (Beyotime, China). The protein was separated by SDS-PAGE, electrotransferred to PVDF membranes, incubated with primary antibodies at 4°C overnight, washed three times, incubated for 1 hour with secondary antibodies at room temperature, and visualized by chemiluminescence reagents. Anti-mouse IL-33 (ab187060), anti-ROR*γ*t (ab207082), and anti-FOXP3 (ab187060) antibodies (mAbs) were purchased from Abcam.

### 2.9. Preparation of a Single Lung Cell Suspension and Flow Cytometric Analysis

The lung was removed from the surrounding tissue, sheared, crushed, and added to the medium containing different concentrations of digestive enzymes (RPMI 1640, DNAse I, and DNAse II) (Invitrogen, USA), and incubated at 37°C. The resulting suspension was passed through a 70-micrometer nylon cell strainer (BD, USA). The samples were centrifuged at 1000 r/min for 5 min at 4°C, washed, and resuspended in PBS after the lysis of RBCs. The following antibodies (eBioscience, USA) were used for surface staining: FITC-labeled anti-CD4 (11-0041-82), PE-labeled anti-IL-17 (61-7177-80), APC-labeled anti-FOXP3 (17-5773-80), and PE-labeled anti-CD25 (25-0251-81). To analyze Th17 cells, the cells were stimulated with a Cell Activation Cocktail (BioLegend, USA) and then incubated with FITC-labeled anti-CD4, followed by fixation and permeabilization using a Cytofix/Cytoperm kit (BD Biosciences, USA). Intracellular cytokine labeling was performed with PE-labeled anti-IL-17 according to the manufacturer's instructions. To analyze Treg cells, the cells were incubated with FITC-labeled anti-CD4 and PE-labeled anti-CD25 antibodies, followed by a similar experimental procedure as stated above. Cell marker expression analysis was performed using a Navios instrument (Beckman Coulter, USA). Data were analyzed with the FlowJo software.

### 2.10. Administration of rmIL-33

rmIL-33 (3236-ML-010; R&D Systems) was administered via an intraperitoneal injection of 1000 ng per mouse. IL-33 administration was performed on day 0 following intratracheal instillation of LPS or PBS.

### 2.11. Bone Marrow-Derived Dendritic Cell (BMDC) Generation

Mouse BMDCs were generated as previously described [20]. The femurs and tibiae of C57BL/6 mice were cut, and the marrow was flushed with ice-cold PBS using a syringe. Red blood cells were lysed with lysing solution containing 0.15 M NH_4_Cl, 1 mM KHCO_3_, and 0.1 mM EDTA. The number of bone marrow cells was adjusted to 0.5‐1 × 10^6^ cells/well (1 ml), which were added to 24-well plates and cultured in RPMI 1640 medium containing 10% FBS (Gibco) with GM-CSF (20 ng/ml) and IL-4 (10 ng/ml) (Sigma-Aldrich). Half of the medium was refreshed, and the medium containing these factors was added every two days. On day 7, loosely adherent DC aggregates were harvested for use in the experiments. LPS (1 *μ*g/ml) was added and incubated for 24 h to induce the maturation of BMDCs. The supernatants were collected, and IL-6 and IL-23 levels were measured by ELISA. All cell cultures were maintained at 37°C in a humidified atmosphere of 5% CO_2_ and 95% air.

### 2.12. Statistical Analysis

All data are expressed as the means ± standard deviation (SD) and were analyzed with GraphPad Prism 8 (GraphPad Software, La Jolla, California USA). Comparisons of continuous variables among multiple groups were performed with one-way ANOVA followed by Bonferroni's post hoc test. Comparisons of continuous variables distributed between two independent groups were performed with unpaired Student's *t*-tests. Significant differences are shown as ^∗^*P* < 0.05, ^∗∗^*P* < 0.01, and ^∗∗∗^*P* < 0.001.

## 3. Results

### 3.1. LPS Challenge Increased the Expression of IL-33 in Mice

**Figure 1:**
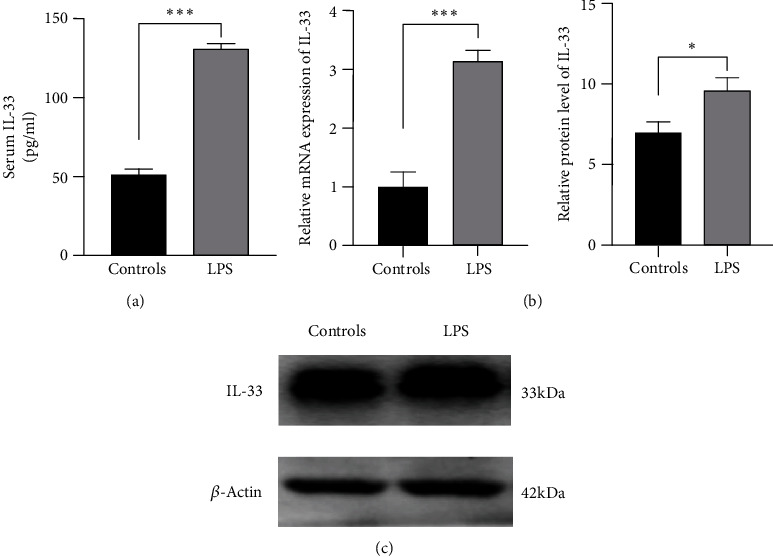
Elevated expression of IL-33 in mice with ARDS. (a) The protein level of IL-33 in peripheral blood was measured by ELISA. (b) The mRNA expression of IL-33 in the lung was measured by qRT-PCR. (c) The level of IL-33 protein in lung tissue was measured by Western blotting. The data are expressed as the means ± SDs. Significant differences are shown by ^∗^*P* < 0.05, ^∗∗^*P* < 0.01, and ^∗∗∗^*P* < 0.001 by unpaired Student's *t*-tests comparing the normal WT controls and ARDS groups. *N* = 5 for each group, and three independent repeated experiments were carried out.

In comparison to the normal WT controls, LPS-challenged mice had a much higher concentrations of serum IL-33 at 48 hours (Figure 1(a)). The mRNA level and protein expression of IL-33 in the lung tissue of challenged mice increased significantly relative to that in the normal WT controls (Figures 1(b) and 1(c)).

LPS challenge increased the expression of IL-33 at the peak of pathological damage in mice, suggesting that IL-33 might play an important role in the pathogenesis of ARDS.

### 3.2. IL-33 Deficiency Improved the Pulmonary Histopathology of Mice with LPS-Induced ARDS

**Figure 2:**
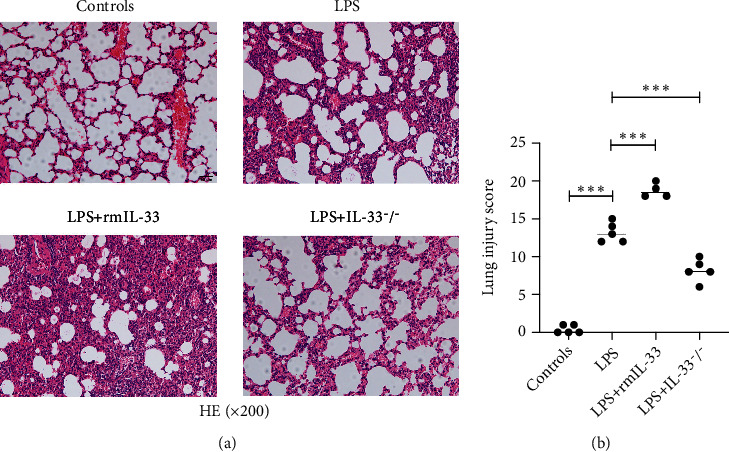
IL-33 deficiency alleviated pulmonary histopathology in mice with ARDS. (a) Representative images of HE staining of lung sections; the magnification of histological images is ×200. (b) Lung injury scores were measured by the Smith method. The data are expressed as the means ± SDs. Significant differences are shown by ^∗^*P* < 0.05, ^∗∗^*P* < 0.01, and ^∗∗∗^*P* < 0.001 by one-way ANOVA followed by Bonferroni's post hoc test comparing the WT control, LPS, LPS+rmIL-33, and LPS+IL-33^−/−^ groups. *N* = 3‐5 for each group, and three independent repeated experiments were carried out.

IL-33-deficient mice were used to examine the role of IL-33 in the context of LPS-induced experimental ARDS. After LPS administration, pulmonary histopathology revealed thickening of the alveolar septum, apparent neutrophil infiltration in the alveolar and interstitial space, hemorrhage, and alveolar exudate in the lung tissues of mice in the LPS group (Figure 2(a)), and the Smith score for quantifying lung injury was also increased significantly (Figure 2(b)) in the LPS group. However, LPS+IL-33^−/−^ mice exhibited alleviated histopathologic characteristics and lower Smith scores than WT mice in the ARDS group (Figures 2(a) and 2(b)).

To further verify the effect of IL-33, exogenous rmIL-33 was injected intraperitoneally following intratracheal instillation of LPS. Histological evaluations showed that the administration of rmIL-33 led to more severe lung injuries than those in the WT mice in the LPS group (Figures 2(a) and 2(b)).

These results indicated that IL-33 deficiency had a protective effect on pulmonary histopathology in LPS-induced ARDS, given the exacerbating effect of exogenous rmIL-33.

### 3.3. IL-33 Deficiency Inhibited Lung Inflammation in Mice with LPS-Induced ARDS

**Figure 3:**
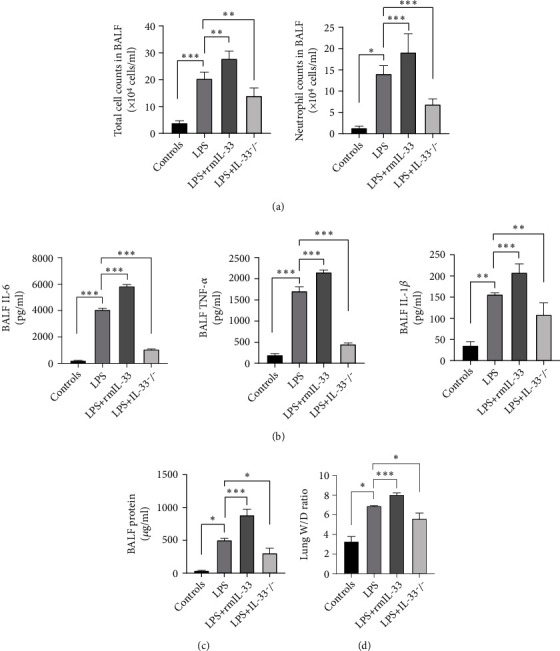
IL-33 deficiency diminishes pulmonary inflammatory responses in mice with ARDS. (a) Total cell counts and neutrophil counts in BALF. (b) The expression of inflammatory mediators, including TNF-*α*, IL-1*β*, and IL-6, in BALF was detected by ELISA. (c) Pulmonary permeability was assessed by BALF protein analysis. (d) Lung tissue edema was measured by the lung W/D ratio. The data are expressed as the means ± SDs. Significant differences are shown by ^∗^*P* < 0.05, ^∗∗^*P* < 0.01, and ^∗∗∗^*P* < 0.001 by one-way ANOVA followed by Bonferroni's post hoc test comparing the WT control, LPS, LPS+rmIL-33, and LPS+IL-33^−/−^ groups. *N* = 3‐5 for each group, and three independent repeated experiments were carried out.

To assess inflammation in the lungs, the cell counts and concentrations of the proinflammatory cytokine TNF-*α*, IL-1*β*, and IL-6 in BALF were analyzed by ELISA. To assess pulmonary permeability, the protein in BALF and the W/D ratio were determined. After LPS challenge, total cell and neutrophil counts in BALF were markedly increased in the LPS group (Figure 3(a)). The expression of TNF-*α*, IL-1*β*, and IL-6 in BALF was also increased in the LPS group (Figure 3(b)). The protein levels in BALF and the W/D ratio were higher in the LPS group than the control group (Figures 3(c) and 3(d)). Indications of LPS-induced lung injury, including pulmonary histopathology, protein levels in BALF, the wet/dry ratio, total cell counts and neutrophil counts in BALF, and the levels of the proinflammatory cytokines TNF-*α*, IL-1*β*, and IL-6, demonstrated that our murine model of ARDS represents a powerful experimental tool to further investigate the pathologic processes of LPS-induced ARDS.

Compared with those in the LPS group, the cell counts and levels of TNF-*α*, IL-1*β*, and IL-6 were significantly decreased in the LPS+IL-33^−/−^ group (Figures 3(a) and 3(b)) but were significantly increased in the LPS+rmIL-33 group. The same changes in protein levels in BALF and the W/D ratio were also observed in the LPS+IL-33^−/−^ group and the LPS+rmIL-33 group (Figures 3(c) and 3(d)).

### 3.4. IL-33 Deficiency Inhibited Th17 Cell Development and Weakened Th17 Cell Function in Mice with ARDS

**Figure 4:**
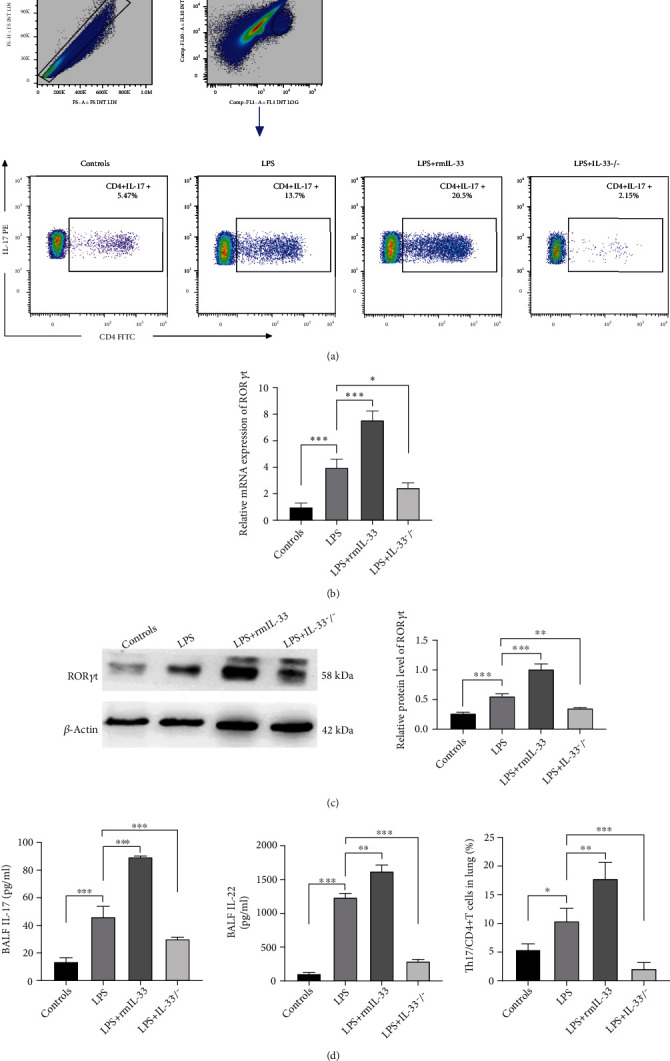
IL-33 deficiency inhibited Th17 cells in murine ARDS. (a) The percentage of CD4+ IL-17+CD4+ T cells in the lung was detected by flow cytometry. (b) The mRNA expression of ROR*γ*t in the lung was measured by real-time PCR. (c) The protein expression of ROR*γ*t in lung tissue was measured by Western blotting. (d) The concentrations of IL-17 and IL-22 in BALF were detected by ELISA. The data are expressed as the means ± SDs. Significant differences are shown by ^∗^*P* < 0.05, ^∗∗^*P* < 0.01, and ^∗∗∗^*P* < 0.001 by one-way ANOVA followed by Bonferroni's post hoc test comparing the WT control, LPS, LPS+rmIL-33, and LPS+IL-33^−/−^ groups. N = 3‐5 for each group, and three independent repeated experiments were carried out.

To confirm whether IL-33 could impact the Th17 cell population in ARDS lungs, we analyzed cells by flow cytometry. The proportion of Th17 cells was significantly increased in the LPS+rmIL-33 group compared to the LPS group but was significantly decreased in the LPS+IL-33^−/−^ group (Figure 4(a)). IL-33 deficiency decreased the percentage of Th17 cells in the lung, which was accompanied by decreased mRNA and protein expression of ROR*γ*t (Figures 4(b) and 4(c)). The administration of rmIL-33 reversed the effects on these parameters (Figures 4(b) and 4(c)).

To verify the effects of IL-33 on Th17 cell function, we further measured the levels of the functional cytokines IL-17 and IL-22. The results showed that the administration of rmIL-33 increased the concentrations of IL-17 and IL-22 in BALF. However, IL-33 deficiency attenuated these changes in expression levels (Figure 4(d)).

Collectively, IL-33 deficiency inhibited Th17 cells in murine ARDS.

### 3.5. IL-33 Deficiency Improved the Th17/Treg Imbalance in Mice with ARDS

**Figure 5:**
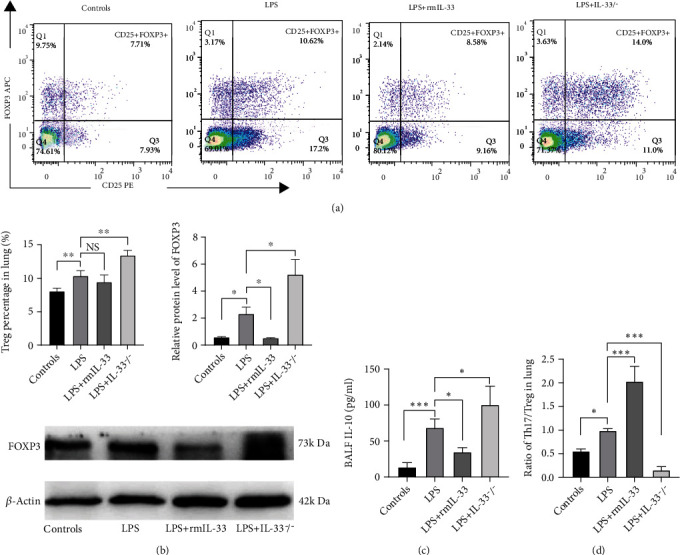
IL-33 deficiency enhanced the Treg cell response in murine ARDS. (a) The percentage of FOXP3+CD25+CD4+ T cells in the lung was detected by flow cytometry. (b) FOXP3 protein expression in lung tissue was measured by Western blotting. (c) The concentrations of IL-10 in BALF were detected by ELISA. (d) The ratio of Th17/Treg cells in the lung. The data are expressed as the means ± SDs. Significant differences are shown by ^∗^*P* < 0.05, ^∗∗^*P* < 0.01, and ^∗∗∗^*P* < 0.001 by one-way ANOVA followed by Bonferroni's post hoc test comparing the WT control, LPS, LPS+rmIL-33, and LPS+IL-33^−/−^ groups. *N* = 3‐5 for each group, and three independent repeated experiments were carried out.

Considering that Th17 and Treg cells are paired CD4+ T cell subsets and that Th17/Treg cells play a vital role in the inflammatory process of ARDS, we next examined the effect of IL-33 deficiency on Treg cell immune responses, which are thought to be anti-inflammatory. LPS-treated mice had increased Treg cell percentages, FOXP3 protein levels, and related cytokine IL-10 levels in BALF. There were increases in the Treg cell percentage, FOXP3 protein levels in the lung, and IL-10 levels in the BALF of LPS-treated mice (Figures 5(a)–5(c)). IL-33 deficiency enhanced the Treg cell response by increasing the Treg/CD4+ T cell percentage and FOXP3 protein levels in the lung and IL-10 levels in the BALF of mice with ARDS. The Th17/Treg ratio in the LPS+IL-33^−/−^ group was significantly lower than that in the LPS group and was similar to that in the WT control group (Figure 5(d)). These results suggested that IL-33 deficiency could regulate the immune response of Th17 and Treg cells and improve the imbalance in Th17/Treg cells in the lungs of mice with ARDS.

### 3.6. IL-33 Deficiency Impaired Th17 Differentiation and Impaired DC-Derived IL-6 and IL-23 Production

**Figure 6:**
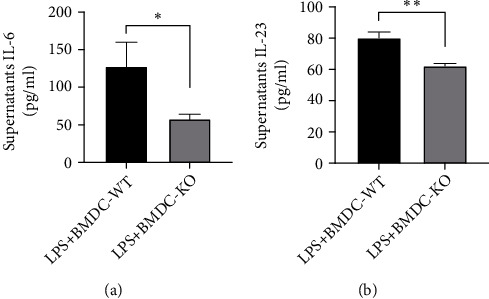
IL-33 deficiency impairs IL-6 and IL-23 production by BMDCs. BMDCs were induced and stimulated with LPS, and the concentrations of IL-6 (a) and IL-23 (b) in the culture supernatant were measured by ELISA. The data are expressed as the means ± SDs. Significant differences are shown by ^∗^*P* < 0.05, ^∗∗^*P* < 0.01, and ^∗∗∗^*P* < 0.001 by unpaired Student's *t*-tests comparing the WT and IL-33-KO groups. *N* = 3 for each group, and three independent repeated experiments were carried out.

To gain insight into the mechanisms by which IL-33 deficiency mediates the Th17 cell differentiation, bone marrow was isolated from C57BL/6 and IL-33^−/−^ normal mice and then cultured in complete RPMI 1640 for 7 days to obtain BMDCs. IL-6, which initiates the development of Th17 cells and forms a positive feedback loop with IL-17A [21], is a key cytokine for the differentiation of Th17 cells from naïve CD4+ T cells [22]. IL-23 plays a role in stimulating IL-17 production by T cells during inflammation [23]. In addition, the expression of FOXP3 is reduced when IL-6 is added to naïve CD4+ T cells [24]. BMDCs were seeded in 24-well plates, the supernatants were collected, and cytokines, including IL-6 and IL-23, were measured. The level of IL-6 secreted by IL-33^−/−^ DCs was decreased compared with that in the WT control group (Figure 6(a)). IL-23 also decreased significantly in IL-33^−/−^ DCs (Figure 6(b)).

## 4. Discussion

The pathogenesis of ARDS is a clinical syndrome characterized by the dysfunction of pulmonary epithelial and capillary endothelial cells, the infiltration of alveolar macrophages and neutrophils, cell apoptosis, necroptosis, and fibrosis [25]. It also involves different immune cells that mediate the propagation of lung injury triggered by a direct pulmonary or indirect systemic insult [26]. Therefore, the relationship between the molecular regulation of immune cells and the pulmonary microenvironment may offer a strong foundation for developing therapeutic interventions. In this study, we demonstrated that (1) IL-33 deficiency ameliorated lung histopathology of lung tissue and decreased inflammatory cytokine levels in mice with ARDS. (2) IL-33 deficiency could significantly modulate the differentiation and function of Th17 cells. (3) IL-33 deficiency significantly impacted the Th17/Treg ratio. (4) The effect of IL-33 on Th17 and Th17/Treg balance may be mediated by DCs.

IL-33 is mainly expressed by epithelial cells, endothelial cells, and fibroblasts in barrier tissues as an important cytokine associated with barrier defense, immune regulation, tissue repair, and metabolic homeostasis [27–29]. Previous studies have shown that IL-33 can be released by damaged or necrotic barrier cells (endothelial and epithelial cells) and act as an alarmin [30]. In this study, we found that mice with LPS-induced ARDS had not only higher levels of IL-33 in BALF but also elevated lung mRNA and protein expression of IL-33 compared to normal WT controls. This finding indicated that IL-33 could be a major factor in ARDS and may be correlated with subsequent immune responses, which was consistent with the previous findings [31].

Emerging evidence identified the immunomodulatory function of IL-33 in many infectious diseases [32–34]. However, among them, IL-33 has been shown to be a Janus cytokine in infectious disease mechanisms [35–38]. IL-33 can be protective during pneumonia-induced acute lung injury [36], whereas it contributes to early inflammation-associated lung injury during abdominal sepsis [37]. Our study showed that IL-33 gene depletion decreased lung permeability and inflammatory cytokine levels and improved the histopathology of lung tissue in mice with ARDS. Notably, exogenous rmIL-33 exacerbated lung immunopathology in mice with ARDS. These results indicated that IL-33 deficiency could improve the inflammatory state of ARDS in animal models. The pleiotropic function of IL-33 might be due to different triggering insults, different infection stages, different immune cell types, and related cytokines [36, 38–40]. Therefore, we further investigated the detailed molecular mechanisms by which IL-33 deficiency improves the pathology of mice with ARDS.

T cell immunity, which is an important part of adaptive immunity, is involved in the development of ARDS [41]. In a series of animal model studies, it has been suggested that suppressing the Th17 response could improve the immunity and inflammatory state of ARDS [10, 41, 42]. The mechanism mainly involves the capacity to produce abundant inflammatory cytokines, including IL-17 and IL-22 [43], and the recruitment of neutrophils in the lung [44]. Treg cells exert immunosuppressive effects by secreting anti-inflammatory cytokines such as IL-10. Moreover, the relationship between Th17 cells and Treg cells is inseparable, and Th17 cells can influence the immunosuppressive effect of Treg cells [45]. The Th17/Treg balance is critical in maintaining immune homeostasis, and restoring the Th17/Treg balance can reduce inflammation in ALI [46]. Recent experimental evidence revealed that IL-33 plays a multifaceted role in the development and maintenance of the Th17 immune response. IL-33 may control proinflammatory Th17 cells in the small intestine, and proinflammatory Th17 cells acquire a regulatory phenotype with immunosuppressive properties in response to IL-33 [47]. In a model of *Citrobacter rodentium*-driven infectious colitis, IL-33 inhibited the differentiation of IL-17A-producing CD4+ T cells, which have the potential to reverse the negative effect of IL-33 during infection [34]. On the other hand, IL-33 skews Th17 cell responses in lymphoid organs by converting Treg cells to Th17 cells and promoting Th17 cell differentiation [15, 20]. These experiments provide further evidence that IL-33 influences the function of Th17 cells and that this effect is highly dependent on the target tissue of infection and type of pathogen. Our results showed that the frequencies of lung Th17 cells were decreased in IL-33^−/−^ mice with ARDS relative to WT control mice, which was consistent with the decreases in the mRNA expression and protein levels of the corresponding biomarker ROR*γ*t. In addition, the cytokine production profile revealed that IL-33 deficiency decreased IL-17 and IL-22 levels. In contrast, exogenous rmIL-33 increased the frequencies of lung Th17 cells, along with the expression of ROR*γ*t and the levels of IL-17 and IL-22 in BALF. The frequencies of lung Treg cells and the levels of FOXP3 and IL-10 in IL-33^−/−^ mice with ARDS were increased. These results suggest that a decrease in the Th17 immune response and a reduction in the ratio of Th17/Treg cells are associated with the protective effect of IL-33 deficiency on ARDS.

Next, we investigated the mechanism by which IL-33 induced the Th17 cell response. In autoimmune disease, self-reactive Th17 cells differentiate from naïve CD4+ T cells via self-antigens presented by DCs in lymph nodes [15]. DCs influence the fate of naïve CD4+ T cells by producing cytokines such as IL-6, IL-1, and IL-23 [48]. DCs are activated by IL-33 signaling to orchestrate adaptive immune responses, inducing the differentiation of Th17 cells via DC maturation [20, 28]. Thus, we hypothesized that IL-33 may induce the differentiation of Th17 cells via DCs in the presence of antigens. IL-6 is the key cytokine required to induce the Th17 phenotype and is sufficient for the induction of IL-17 expression in naïve CD4+ T cells due to reduced FOXP3 expression [24]. IL-23 also plays a role in stimulating IL-17 production by T cells in inflammation [23]. In this study, we measured the levels of IL-6 and IL-23 during the LPS activation of DCs and found that the levels of IL-6 and IL-23 were markedly decreased in IL-33-deficient DCs. Taken together, IL-33 deficiency may suppress the differentiation of Th17 cells and restore the Th17/Treg cell balance via IL-6 and IL-23 secreted by DCs.

## 5. Conclusion

In summary, IL-33 deficiency could attenuate lung injury and regulate lung inflammation by suppressing the Th17 immune response and impacting the Th17/Treg cell balance in mice with ARDS.

## Figures and Tables

**Table 1:** Primer sequences of target mRNAs.

Gene	Forward (5′⟶3′)	Reverse (5′⟶3′)
IL-33	TAACACAGTCTCCTGCCTCCC	CACACCGTCGCCTGATTGA
ROR*γ*t	CGCACCAACCTCTTTTCACG	CGACTTCCATTGCTCCTGCT
GAPDH	TGTGTCCGTCGTGGATCTGA	TTGCTGTTGAAGTCGCAGGAG

## Data Availability

The raw/processed data required to reproduce these findings cannot be shared at this time as the data also forms a part of an ongoing study.
